# Adipogenesis Regulation and Endocrine Disruptors: Emerging Insights in Obesity

**DOI:** 10.1155/2020/7453786

**Published:** 2020-02-18

**Authors:** Jorge Enrique González-Casanova, Sonia Liliana Pertuz-Cruz, Nelson Hernando Caicedo-Ortega, Diana Marcela Rojas-Gomez

**Affiliations:** ^1^Instituto de Ciencias Biomédicas, Facultad de Ciencias de la Salud, Universidad Autónoma de Chile, Santiago, Chile; ^2^Programa de Nutrición y Dietética, Departamento de Nutrición Humana, Facultad de Medicina, Universidad Nacional de Colombia, Bogotá, Colombia; ^3^Departamento de Ingeniería Bioquímica, Facultad de Ingeniería, Universidad ICESI, Cali, Colombia; ^4^Escuela de Nutrición y Dietética, Facultad de Medicina, Universidad Andres Bello, Santiago, Chile

## Abstract

Endocrine disruptors (EDs) are defined as environmental pollutants capable of interfering with the functioning of the hormonal system. They are environmentally distributed as synthetic fertilizers, electronic waste, and several food additives that are part of the food chain. They can be considered as obesogenic compounds since they have the capacity to influence cellular events related to adipose tissue, altering lipid metabolism and adipogenesis processes. This review will present the latest scientific evidence of different EDs such as persistent organic pollutants (POPs), heavy metals, “nonpersistent” phenolic compounds, triclosan, polybrominated diphenyl ethers (PBDEs), and smoke-derived compounds (benzo -alpha-pyrene) and their influence on the differentiation processes towards adipocytes in both *in vitro* and *in vivo* models.

## 1. Introduction

The prevalence and incidence of overweight and obesity worldwide have increased significantly in the last three decades. According to the World Health Organization (WHO), since 1975, obesity has nearly tripled, and every year, at least 2.8 million people die as a result of obesity or overweight. By 2016, 39% of adults were overweight, and 13% were obese; 41 million children under 5 years of age and 340 million children and adolescents between 5 and 19 years of age were overweight or obese [1]. These figures explain the reason why this disease has reached epidemic proportions, and its understanding and intervention are public health priorities.

Diverse studies indicate that the etiology of this chronic disease is multivariate and complex. The predisposing biological factors include genetic characteristics, prenatal determinants, pregnancy, menopause, intestinal microbiota, and viruses. People prone to develop obesity may also be affected by behavioral causes such as excessive energy intake, increased portion sizes, and the practice of a sedentary lifestyle. On the contrary, genetic predisposition to obesity can be influenced by epigenetic triggers such as high availability of food, socioeconomic status, or the presence of chemical contaminants in the environment that could be ingested [2–4].

Due to the deleterious effect of endocrine disruptors on health, it is necessary to characterize the damage by specific dietary exposures of these compounds. This fact implies determining the mechanisms involved in which the food acquires the contaminant. It is also necessary to understand the role as endocrine disruptor and the different physiological and pathological consequences, in particular the relationship with adipogenesis processes.

## 2. Adipogenesis

In living multicellular organisms, certain cell types have the capacity to originate new and specialized cellular lineages, and this characteristic is called cellular differentiation. Cellular differentiation is mainly carried out in embryonic development; however, some tissues retain this property of cell differentiation even when the organism is in an adulthood state.

Cell differentiation is activated by a series of signals at the organism level, where these are then translated into specific processes such as differential expression of genes, activation, or inactivation of transcription factors and cellular signaling proteins. The differentiated cell undergoes a series of morphological changes and arrest of cell growth; however, the genetic material of the cell remains unchanged.

Adipogenesis or adipocyte differentiation has been the focus of many studies in recent years. In this process of differentiation, a mesenchymal stem cell (MSC) has the ability to produce mature adipocytes, which are the main constituents of adipose tissue [5, 6].

Adipose tissue was formerly described as a nondynamic tissue whose function was limited to constitute the energy reservoir of the organism, through the accumulation of triglycerides. From the 90s, this tissue begins to attract the attention of scientists, and it is now considered as a highly active and dynamic tissue with a variety of hormonal, immunological, and regulatory functions of energy homeostasis [7, 8]. This is one of reasons why the research of adipocyte differentiation process has acquired great importance, and also due to its relationship with different pathologies such as obesity, diabetes mellitus type II (DM type II) [9], insulin resistance [10], osteoporosis [11, 12], rheumatoid arthritis, and osteoarthritis [13]. On the contrary, MSCs are multipotential cells that can give rise to different cell lineages such as osteoblasts, chondrocytes, myocytes, and adipocytes [14].

The process of adipocyte differentiation occurs throughout the different stages of development of organisms and is controlled by both nutritional factors as well as by genetic and environmental factors. There are different models of cell lines that are widely used by researchers [15, 16] to study the formation of adipocytes and to understand their relationship with obesity. *In vitro* models highlight the use of mouse embryonic cell lines 3T3-L1 and 3T3-F442A that can be induced to differentiate into adipocytes under chemical and hormonal exposure.

In response to an extracellular signal, an MSC undergoes processes of proliferation and clonal expansion that originate preadipocytes and high plasticity cells, which can be ultimately differentiated into a cell with a characteristic-defined phenotype, the mature adipocyte. In the first stage of the process, MSC converges into a preadipocyte that does not differ morphologically or phenotypically from its precursor cell, but in which activation processes that involve transcription factors of the AP1 family occur. Subsequently, the terminal differentiation stage is initiated where the resulting adipocyte acquires the specialized equipment for the secretion and synthesis of proteins and lipids specific to the lineage to which it has differentiated [17].

Different signals that influence adipogenesis have been described, for example, fibroblast growth factor type 1 (FGF1) [18] and insulin-like growth factor type 1 (IGF1) [19] are known for their induction action. On the contrary, there is an inhibitory effect on adipogenesis when the WNT signaling [20] or the hedgehog pathway [21] are activated.

## 3. Transcription Factors That Regulate Adipogenesis

It is known that the differentiation of adipocytes is a complex process consisting of several stages, and it is widely regulated by both the specific expression of proteins and transcription factors. In the initial phase, adipogenesis is induced by the expression of binding proteins CCAAT/enhancer *β* (C/EBP*β*) and C/EBP*δ*. The activity of these proteins give rise to a second stage since among their targets are the promoters of the genes that code for peroxisome proliferator-activated receptor gamma (PPAR*γ*) and C/EBP*α*. PPAR*γ* is considered the master transcription factor of the adipocyte differentiation process since when it is activated by binding to its ligand, morphological changes and the expression of all genes specific for mature adipocytes are induced [22].

PPAR*γ* plays an essential role in the process of adipocyte differentiation of white and brown adipose tissue. Two PPAR isoforms have been described, which are produced by alternative splicing. The isoform 2 of PPAR, expressed mainly in adipose tissue and whose function is to promote the storage of triglycerides [23], has been related to obesity, insulin resistance [24], and dyslipidemia [25]. PPAR isoform 1 is ubiquitous in other cell types besides adipocytes. PPAR*γ* activates the promoter of gene coding for C/EBP*α*, and reciprocally and inversely, C/EBP*α* activates the PPAR*γ* promoter, generating a positive feedback loop. Both genes cooperate by binding to sites of promoter regions of various genes that are expressed during the differentiation process, as well as in the mature adipocyte [26]. Some examples of these genes are those that code for proteins involved in insulin sensitivity, lipolysis, and lipogenesis. Krox20 [27] and Kruppel-like factors (KLF) [28] have also been reported in the regulation of differentiation of adipocytes.

## 4. Endocrine Disruptors

Recently, many researchers have focused their interest on so-called EDs or obesogens, bringing attention to their possible etiological incidence of obesity. Both experimental and epidemiological evidence support the idea that low doses of chemical contaminants have endocrine and metabolic effects. Most of these contaminants are present in the food chain and accumulate in the fat mass after absorption [29, 30]. Secular evidence suggests that some of these EDs may be involved in the global epidemic of obesity, diabetes (diabetogens), as well as in hormone-dependent cancer [3, 4, 31].

The EDs, are a particular group of well-differentiated chemical compounds, defined as “substances exogenous to the organism that alter the function or functions of the endocrine system, being able to cause adverse effects on the health of an organism, its descendants, in the population in general or in a particular subpopulation” [32]. These substances were initially synthesized to fulfill specific functions, such as the control of pests in agriculture, improve the stability of body lotions, or be part of the structure of certain plastics, but with the time, there have been discovered harmful effects derived from continued exposure to them [33].

Among the characteristics of the obesogens is that they are compounds with very different chemical structures capable of acting at shallow doses, show different mechanisms of action, and be able to alter the hormonal balance [34]. They interfere with the body's ability to regulate growth, its development, metabolism, and other functions. There are hundreds of disruptors in the environment, in food, and everyday products. These can contribute to a variety of diseases and disabilities such as obesity, cancer, diabetes, heart disease, reproductive, or neurodevelopmental problems [34].

The Center for Biomedical Research in Network-Physiopathology of Obesity and Nutrition (CIBERobn), which brings 24 Spanish research groups together, has shown that certain synthetic chemical compounds present in the environment and daily life, associated with pesticides and insecticides, but also perfumes, plastics, or cosmetics, predispose to obesity. These chemical compounds, given their effect on fat gain and obesity [34], are also found in synthetic fertilizers, electronic waste, and food additives that are present in the food chain and products of regular consumption such as food, beverages, personal care products, and household cleaning products [35–37].

The possible causality between this group of compounds and the overweight and obesity etiology has been investigated since the increase in the prevalence of obesity. Other metabolic diseases have been associated with the increase in exposure to EDs, as well.

## 5. Obesogenic Compounds and Adipogenesis

**Figure 1:**
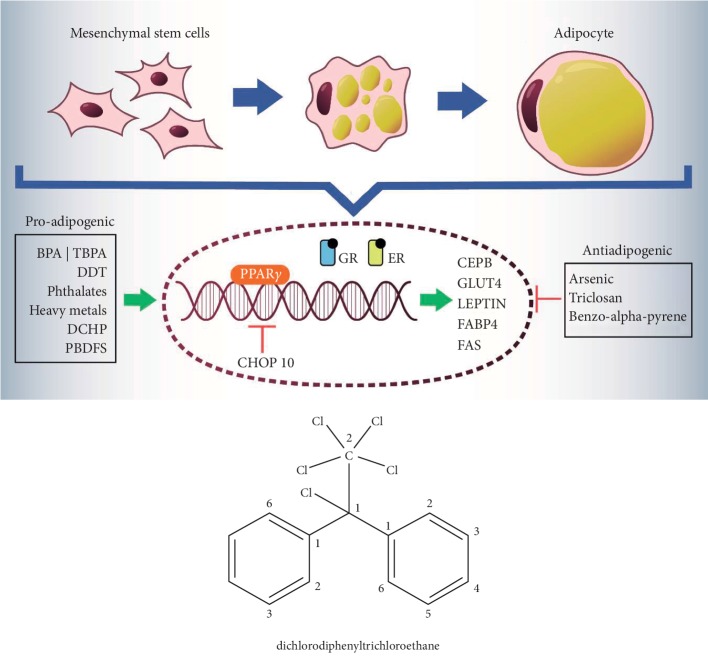
Schematic representation of effects of endocrine disruptors on adipogenic differentiation.

As previously mentioned, the obesogenic compounds are heterogeneous and come from various sources (Table 1). So far, there is the characterization of some of those and their effects on the processes of differentiation towards adipocyte [42–44] (Figure 1) (Table 2):

### 5.1. Persistent Organic Pollutants (POPs)

POPs are chemical substances soluble in fats and, therefore, in the organism; they bioaccumulate in fat reserve tissues and biomagnify in the food chain [33, 36]. The most important route of human exposure to POPs is the consumption of food, especially those of animal origin. Some studies have identified the presence of POPs in oils and fats, meat, eggs, milk, and fish from freshwater ecosystems [38, 80–82].

This phenomenon is explained by its high resistance to chemical degradation and, therefore, its great persistence in the environment and living beings. There is growing epidemiological evidence that frequent exposure to low doses of sure POPs may be related to obesity and metabolic pathologies in the predisposed genetic population. Exposure during early pregnancy to pesticides can lead to the development of obesity in childhood and have been associated with diseases such as diabetes, hypertension, dyslipidemia, and BMI [33, 83].

POPs include some pesticides such as dichloro diphenyl trichloroethane (DDT) or hexachlorobenzene (HCB) and some industrial chemicals such as polychlorinated biphenols (PCBs). While the use of these chemicals is prohibited in many countries, their presence persists in the environment due to their high stability, and they are still used in some developing countries [37]. DDT was developed in the 1940s as an insecticide and was also used to combat diseases transmitted by insects, such as malaria. Because DDT has been found to be genotoxic and possibly carcinogenic, its use was banned in the USA in 1972 and the Netherlands in 1973. However, in developing countries, DDT is still used for vector control like malaria [84].

DDT may affect the physiology of adipose tissue. The exposure of DDT in cell cultures has a proadipogenic effect, increasing the expression of PPAR*γ* and the binding of the C/EBP*δ* protein to DNA during adipogenesis [47]. Studies in the NIH3T3-L1 cell line exposed to the pesticide dichlorodiphenyldichloroethylene (DDE) showed no effect on adipogenesis; however, in the presence of DDT, the mature adipocytes expressed more leptin, resistin, and adiponectin. Similarly, Howell and Howell [45] showed that, in human mesenchymal stem cells (hMSC), DDT could significantly increase the process of differentiation into adipocyte and the molecular markers typical of the fat cell, such as PPAR*γ*, leptin, FABP4, and GLUT4 [46]. Additional experimental evidence suggests that the proadipogenic activity of DDT would be through the phosphorylation of the AMPK*α* protein [48].

### 5.2. Heavy Metals

In general, many metals have solubility in organic solvents. Human exposure to certain metals such as arsenic, cadmium, and lead has been associated with metabolic alterations such as an increased risk of suffering from DM type II, cardiovascular disease, and obesity. The accumulation of mercury in large fish growing in contaminated water sources, cadmium in cereals and viscera, lead in tubers, or cadmium and arsenic in vegetables have been widely documented [32, 39].

Regarding the influence of heavy metals in the process of adipogenesis, Beier et al. [85] demonstrated through experiments with rats exposed to low concentrations of lead before conception and for 18 months that this heavy metal could stimulate differentiation in mesenchymal cells to mature adipocytes with a concomitant detriment of osteoblastogenesis. This process was further characterized by an inhibition of the cellular signaling pathway of Wnt/*β*-catenin. More recently, the proadipogenic effect of lead was demonstrated in 3T3-L1 cultures, which involved the activation of the ERK, C/EBP*β*, and PPAR*γ* pathways [86].

Studies regarding adipogenesis in zebrafish with exposure to cadmium showed a positive association of this metal with the accumulation of adiposity [87].

Regarding arsenic and its role in adipogenesis, there is scientific evidence of its inhibitory influence on adipocyte differentiation. Hou et al. [49] exposed 3T3 L1 cells to this metal and discovered that it is capable of inhibiting adipogenesis through the activation of CHOP10, an inhibitory molecule for the transcriptional activity of C/EBP*β*, thus causing the suppression of adipogenesis. CHOP10 is a protein that increases its expression in response to the stress of the endoplasmic reticulum produced by the incorrect folding of proteins. Similarly, Beezhold et al. [50] showed that arsenic can increase the expression of microRNA, miR-29b, involved in the regulation of the cell cycle and in the increase in the expression of cyclin D1, which results in inhibition of the differentiation towards the fat cell.

### 5.3. Other “Nonpersistent” Phenolic Compounds

Denotes a wide variety of chemical compounds used in industrial applications, with the main characteristic that they suffer a relatively rapid degradation and/or excretion in the body. In spite of this, constant exposure provokes the continual presence of them in biological samples of the general population [32]. Within this group, it is possible to find the bisphenol A (BPA).

BPA is a “nonpersistent” phenolic compound widely used in the manufacture of polycarbonate plastics and epoxy resins. It is present in the linings of canisters, specific plastic containers, thermal printing papers, dental composite fillings, medical devices, polycarbonate, plastic resins, and materials used in food containers among others. It is highly elastic and resistant to heat material [29]. It has been shown that BPA can migrate from food containers and contaminate them, so these can be an important source of exposure to this compound.

The majority of the population is exposed to BPA daily, and there is currently an unprecedented controversy regarding its possible metabolic disruptor effect since experimental studies have shown that exposure to BPA induces an increase in weight in mice, as well as a high risk of DM type II [32]. Through experiments with rats, it has been demonstrated that relatively low doses of BPA—equivalent to daily and frequent exposure levels in large part of the population—act in a similar way to estradiol, the most potent form of estrogen that, among other aspects, influences the distribution of body fat in women. Exposure to these compounds at inadequate levels and certain stages of development—especially in the fetal stage and childhood—exerts a significant influence on both obesity and the development of diabetes [34]. Several epidemiological studies have found that high urinary concentrations of BPA in adults and children were associated with obesity and increased waist circumference [36].

BPA is a compound that has also been widely studied regarding its effect on lipid metabolism and cellular processes of adipocyte differentiation. It has been associated that a high intake of BPA together with a high-fat/high-sucrose diet leads to similar changes in the structure of the intestinal microbial community in mice [35]. Studies in humans have shown that prenatal exposure to BPA is associated with an increase in body fat at 7 years of age or an increase in body mass index at 9 years of age [36]. Regarding the effect of prenatal exposure to BPA, it has been shown that in experimental models with animals, there is an increase in food consumption, body weight, adipose tissue, and decrease in adiponectin concentrations [51, 52, 59]. These results support the hypothesis that exposure to BPA in critical states of adipose tissue development may alter the homeostasis of the adipocyte, thus increasing the risk of developing complications related to obesity [88].

Regarding the influence of BPA on adipogenesis, several studies have shown this correlation. Using cell models of the 3T3-L1 line exposed to BPA, it was observed that the process of adipogenesis is exacerbated [53–55, 89]. In this same line of evidence, Chamorro-García et al. [56] stimulated 3T3 L1 cells with different concentrations of BPA, and at 10 nM, adipogenesis was stimulated. At 100 nM, a significant increase in triglyceride accumulation was observed. Chronic exposure to BPA in cultures of 3T3-L1 preadipocytes for three weeks at concentrations of 1nM increased the proliferation of preadipocytes and produced hypertrophic adipocytes with impaired insulin signal, reducing glucose utilization and increasing the production of proinflammatory interleukins [57].

The target molecule of BPA's action that would play a significant role in this process is not fully elucidated yet; however, some research groups would bet on the master regulator of adipogenesis: PPAR*γ*. Phrakonkham et al. [58] demonstrated that concentrations of 80 *μ*M BPA are capable of activating PPAR*γ* and adipocyte-specific proteins such as leptin, FAS, and C/EBP*β*. Exposure of BPA in prenatal conditions can increase the expression of PPAR-*γ* in the liver when compared with the control group or with animals fed high-fat diets [60]. Watt and Schlezinger [61] demonstrated a proadipogenic and antiosteogenic effect in mesenchymal stromal cells of bone marrow through the activation of this transcription factor.

Studies aimed to evaluate the adipogenesis *in vivo* demonstrated that direct exposure of BPA in rats through placenta and milk increases adipogenesis in a sex-specific manner since they observed an increase in proadipogenic transcripts such as PPAR*γ*, C/EBP-*α*, and sterol regulatory element-binding factor 1 (SREBF-1) [62]. In studies with sheep, gestational exposure to BPA overregulates the expression of PPAR*γ* in females; however, in male sheep, it reduced the expression of PPAR*γ*, showing that the effect of BPA can be sex-specific [63].

Additional studies have attempted to show that BPA alters the processes of adipogenic differentiation in a manner independent of the regulation of PPAR*γ*. Experiments by Chamorro-García et al. [56] show that BPA alone is capable of activating adipogenesis in 3T3 L1 cells; however, it fails to stimulate differentiation in stromal cells. When using PPAR*γ* antagonists in the 3T3 L1 cultures, no effect was observed, concluding that the proadipogenic effect of BPA is independent of PPAR*γ*. Sargis et al. [64] showed through luciferase assays in cultures of 3T3 L1 that BPA may possess intrinsic glucocorticoid-like activity, promoting adipogenesis through potentiation of the glucocorticoid receptor (GR) activity.

Studies on the mechanism of BPA in adipogenesis in preadipocytes of donors with healthy BMI showed induction of adipogenesis in the absence of exogenous glucocorticoids. Estradiol had no positive effect on differentiation, but BPA-induced adipogenesis was inhibited by estrogen receptor (ER) antagonists, but not by GR antagonists, suggesting that BPA acts through a nonclassical ER pathway [65].

Microarray assays performed on human subcutaneous preadipocytes cultures exposed to BPA demonstrated that the pathways involved in promoting adipogenesis would be via SREBF1, the TR/RXR receptor, and the mTOR pathway [66].

Wang et al. [67] analyzed the effect of BPA on omental adipose tissues of children during adipogenesis and observed an increase in the expression of 11*β*-hydroxysteroid dehydrogenase type 1, an enzyme that catalyzes the conversion of cortisone to cortisol, which is a glucocorticoid proadipogenic. It also observed an increase in PPAR-*γ* and lipoprotein lipase.

Regarding tetrabromobisphenol-A (TBBPA), the brominated analogue of BPA, Riu et al. [55] studied the effect of this compound on cell differentiation towards adipocytes in the NIH3T3-L1 cell line and determined that TBBPA presents specific binding with the protein PPAR*γ*, activating it, which ultimately favored the accumulation of triglycerides in the cell. Similar results were also obtained in the studies of Akiyama et al. [68], who first demonstrated the presence of TBBPA in human milk, but also determined the proadipogenic activity of TBBAP and its brominated derivatives in 3T3-L1 cells.

To understand how TBBPA has a regulatory effect on the process of differentiation towards adipocyte, Woeller et al. [69] propose a mechanism in which TBBPA reduces Thy1 levels, which results in the stimulation of adipogenesis through the induction of microRNA-103.

### 5.4. Phthalates

With regard to phthalates, these compounds are poorly biodegradable and highly bioaccumulable in the food chain [33]. They are mainly used as plasticizers and, therefore, are present in a large number of everyday objects, such as plastic food containers and medical devices, including tubes for parenteral feeding [32]. They are also found in dispersants, lubricants, emulsifying agents, perfumes, and nail polishes. It has been established that the highest exposure to phthalates comes from foods that have absorbed the compound from their packaging or the manufacturing process [33, 40]. Due to their solubility, they are concentrated in fatty foods such as dairy products, high-fat meat, mayonnaise, fat, fish, and shellfish [30]. Phthalates form noncovalent interactions and can easily leach into the environment. This property, combined with its widespread use in consumer products, allows exposure to these chemicals in the US population [36]. Exposure to high levels of phthalates has been associated with alterations in thyroid hormone levels, insulin resistance, increased risk of obesity or low fertility, and increase in BMI and waist circumference [32, 38]. This phenomenon is explained because ingested phthalates cause dysregulation of glucose metabolism, insulin resistance, and adipogenesis [35]. Phthalates are metabolized by the body and metabolites are usually excreted in the urine [45].

Phthalate metabolites have been shown to activate PPAR receptors and have antiandrogenic effects that may contribute to the development of obesity. Prenatal exposure of mice to phthalate DEHP led to an increase in body weight, as well as an increase in body fat in male offspring. Similar findings were reported in different studies with different animal models [37].

Regarding phthalates and their effect on adipogenesis, it has been shown that (mono-(2-ethylhexyl) phthalate) (MEPH) favors the formation of adipocytes through the activation of PPAR*γ* [70, 71]. Hao et al. [72, 90] demonstrated that this proadipogenic effect was dose-dependent; however, uterus exposure of mice significantly increased weight and fat mass in the offspring, possibly indicating an effect of MEHP on adipogenesis in alive mice.

Ellero-Simatos et al. [73] tested the obesogenic effect of MEHP, by stimulating cultures of human preadipocyte cells during adipogenesis. This research group also carried out metabonomic analyzes with the nuclear magnetic resonance (1H NMR) technique and transcriptome analysis. The results of these experiments showed that MEHP could increase the expression of at least 12 transcripts related to the PPAR*γ* signaling pathway, in addition to increasing the expression of genes involved with lipid metabolism: glyceroneogenesis, cytosolic phosphoenolpyruvate carboxykinase, as well as reduction in the release of fatty acids.

Additionally, phthalates have also been related to an alteration in osteoblast homeostasis and adipogenesis in the bone marrow. Studies of Chiu et al. [74], in cell cultures of bone marrow stromal cells exposed to different concentrations of MEHP, showed a decrease in differentiation towards osteoblasts and a concomitant increase in adipogenesis.

Benzyl butyl phthalate (BBP) has also been related to a proadipogenic activity, activating PPAR*γ*, favoring the accumulation of lipids in a dose-dependent manner, and also producing an alteration of lipid metabolism, glyceroneogenesis, and fatty acid synthesis in 3T3-L1 cell cultures [75]. The Sonkar group [76] confirmed the proadipogenic role of BBP, but also suggested a model where BBP produces epigenetic alterations involving the increase of lysine 9 of histone 3 (H3K9), which is typically increased in the promoter of PPAR*γ* in mature adipocyte, which was accompanied by an alteration in histone methylation/acetylation due to the presence of BBP in mouse mesenchymal cell lines C3H10T1/2.

Additionally, Sargis et al. [64] investigated the role of dicyclohexyl phthalate (DCHP) during the process of adipogenesis in 3T3-L1 and discovered that this compound is a glucocorticoid receptor activator, which is a critical regulator in the differentiation towards adipocyte. Based on this result, the group suggests that DCHP could have a proadipogenic effect through a synergistic effect with other adipogenic cell signals.

### 5.5. Triclosan

It is a widely used antibacterial agent commonly found in antibacterial soaps, toothpaste, toothbrushes, dental rinses, laundry detergents, kitchen cutting boards, and plastics in furniture, toys, and sporting goods. In 2016, the Food and Drug Administration (FDA) issued rules prohibiting the use of triclosan in hand and body antibacterial products, citing the lack of evidence to support its effectiveness as an antiseptic. So far, the disruptive endocrine effects of triclosan are not well understood. Some studies have shown effects on the endocrine system of animals. Studies in animals show that high levels of triclosan interfere with estrogen, androgen, and thyroid hormone. Children who are exposed to triclosan may also be more likely to develop allergic hyperreactivity [36]. Concerning its influence on adipocyte differentiation, it has been shown that triclosan has an inhibitory effect of adipogenesis in a model with hMSCs, and this antiadipogenic effect was concentration-dependent, decreasing the production of typical markers of the cell fat, such as adiponectin and lipoprotein lipase [77].

### 5.6. Compounds Derived from Smoke (Benzo-Alpha-Pyrene)

It is a polycyclic aromatic hydrocarbon compound and a potent carcinogen, which originates in combustion processes and is present in foods cooked on the grill or the barbecue [32, 41]. No obesogenic effect has yet been associated; however, an antiadipogenic effect has been demonstrated in cell cultures of human preadipocytes, and this effect is mediated through a specific receptor, the aryl-hydrocarbon receptor (AHR) [79].

### 5.7. Polybrominated Diphenyl Ethers (PBDEs)

They are a class industrialized chemicals widely used in the manufacturing processes of many materials and currently used as effective flame retardants in plastics, electronics, automobiles, homes, furniture, textiles, and construction materials. Different studies have discovered the connection between PBDE and food, which informs about the presence of PBDEs in butter, fish, and other foods, as well as other foods that contain animal fats [33].

Pentabrominated diphenyl ether is increasing in the tissues and body fluids of individuals in the USA and can be detected in blood, breast milk, and urine samples from Americans. Diphenyl ether has generally been eliminated in the USA and banned in the European Union. The PBDEs can accumulate in white adipose tissue and are highly lipophilic. These persistent compounds can be released into the blood, especially during weight loss. They have a structure similar to thyroid hormone, and some studies have found that exposure to these can alter the hormonal balance of the thyroid [36]. They have also been implicated in having a proadipogenic effect in 3T3-L1 cell cultures, increasing the accumulation of lipids and the expression of C/EBP*α*, PPAR*γ* during the differentiation process [78].

## 6. Conclusion

Adipogenesis is a highly controlled process and is regulated by physiological and environmental conditions. Humans are constantly and chronically exposed to a variety of endocrine disruptors, some with obesogenic activity, which act at shallow doses and showing different mechanisms of action altering the hormonal balance. The exposure to the obesogens can happen continuously during the different stages of development, and in this context, the perinatal exhibition is relevant because the effects can be permanent in the organism.

Endocrine disruptors can alter lipid metabolism, promote fat accumulation, and interfere with processes such as adipogenesis. There is sufficient evidence in models with cell lines, human mesenchymal cells and in rodents that demonstrate that obesogens can have targets of action key molecules of the process of differentiation towards adipocyte, such as molecules that regulate the initial stages of differentiation, C/BP*β* and C/EBP*δ*, or secondary stage proteins such as PPAR*γ*.

There are diverse compounds—originally from food—that are associated with possible obesogenic effects; however, there is no enough scientific evidence to prove such association. However, it is necessary to understand in more detail the complexity of the mechanisms involved in the differentiation of fat cells and the influence of EDs in the adipogenesis and the etiology of obesity.

## Figures and Tables

**Table 1:** Source and route of exposure in human of endocrine disruptors.

Endocrine disruptor	Source and main route of exposure in human	Reference
Persistent organic pollutants: DDT	Diet (meat, poultry, milk, and fish) and environmental exposition	Srivastava [38], Wong and Durrani [36]
	Used as insecticide for vector control like malaria	
Heavy metals	Work activity and diet with associated industrial activity (water, food, and the environment)	Ferrer [39]
Lead	Large fish growing in contaminated water sources	Arrebola and Gonzales [32]
Cadmium	Cereals and viscera, lead in tubers	Arrebola and Gonzales [32]
Arsenic	Vegetables	Arrebola and Gonzales [32]
“Nonpersistent” phenolic compounds BPA, TBBPA	Linings of canisters, specific plastic containers, thermal printing papers, dental composite fillings, medical devices, polycarbonate, plastic resins and materials used in food containers	Fénichel and Chevalier [29]
	Inhalation or ingestion of dust; by food intake like fish, milk, eggs, meat, meat products, and breast milk	
Phthalates	Used as plasticizers	Mezcua et al. [33] Azeredo et al. [40]
	Dispersants, lubricants, emulsifying agents, perfumes and nail polishes	
	Comes from foods that have absorbed the compound from their packaging or the manufacturing process	
	Particulate matter in the air, water, or skin contact with plastics that contain it; plastic food containers may also contain DEHP	
	Water from sources of discharge that have had contact with polymers	
Triclosan	Used as antibacterial agent	Wong and Durrani [36]
	Antibacterial soaps, toothpaste, toothbrushes, dental rinses, laundry detergents, kitchen cutting boards and plastics in furniture, toys, and sporting goods	
PBDEs	Used as effective flame retardants in plastics, electronics, automobiles, homes, furniture, textiles, and construction materials	Mezcua et al. [33]
	Butter, fish, and other foods, as well as other foods that contain animal fats	
Benzo-alpha-pyrene	Foods cooked on the grill or the barbecue; smoked, roasted or fried at high temperatures foods; oils subjected to repeated heating	Arrebola and Gonzales [32] Franco-Tobón and Ramírez Botero [41]

**Table 2:** Effect of endocrine disruptor on adipogenesis.

Endocrine disruptor (chemical structure)	Experimental model	Biological effect	Reference
Persistent organic pollutants			
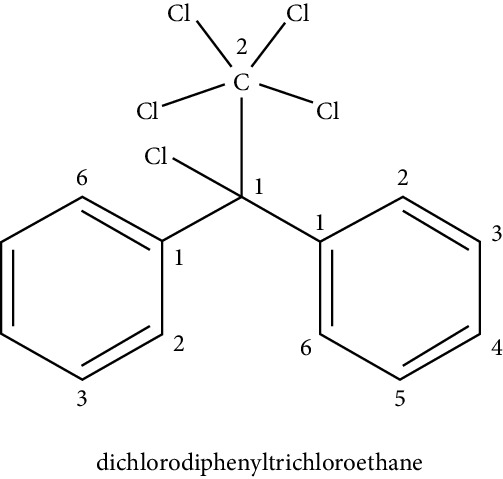	NIH3T3-L1 cells	Proadipogenic effect	Howell and Mangum [45]
	Human mesenchymal cells	Proadipogenic effect, increased expression of PPAR*γ*, leptin, FABP4, and GLUT4	Strong et al. [46]
	3T3 L1 cell culture	Proadipogenic effect, increased expression of PPAR*γ*, and the binding of C/EBP*δ* protein to DNA, increased phosphorylation of the AMPK*α* protein	Moreno-Aliaga and Matsumura [47], Kim et al. [48]

Heavy metals			
Lead	Mesenchymal cells from pregnant rat	Proadipogenic effect, decreased osteoblastogenesis	Hou et al. [49]
		Activation of the ERK pathway and expression of C/EBP*β* and PPAR*γ*	
Cadmium	Zebrafish	Accumulation of adiposity.	Beezhold et al. [50]
Arsenic	3T3 L1 cell culture	Antiadipogenic effect mediated by activation of CHOP10	Angle et al. [51]
	White adipose tissue, human mesenchymal cells	Inhibition of miR-29b activation-mediated adipogenesis	Miyawaki et al. [52]

“Nonpersistent” phenolic compounds			
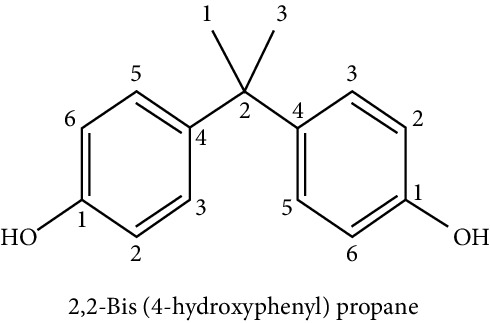	3T3 L1 cell culture	Proadipogenic effect	Masuno et al. [53], Hurst and Waxman [54], Riu et al. [55], Chamorro-García et al. [56], Ariemma et al. [57], Phrakonkham et al. [58]
		Increased expression of PPAR*γ*, leptin, FAS y C/EBP*β*	
		To increase size of the mature adipocyte	
		Increase in insulin resistance, increase in proinflammatory interleukins	
	Prenatal exposure in a murine model	Increase in food intake, increase in body weight, and adipose tissue	Angle et al. [51], Miyawaki et al. [52], Wei et al. [59], García-arevalo et al. [60]
		Increased expression of PPAR-*γ* in liver similar to animals with high-fat diets	
	Bone marrow mesenchymal stromal cells.	Decrease of adiponectin	Watt and Schlezinger [61]
		Proadipogenic and antiosteogenic effect	
	Direct exposure of BPA in placenta and milk in rats	Increase in proadipogenic transcripts such as C/EBP-*α*, PPAR*γ*, and SREBP-1C	Somm et al. [62]
		Increase adipogenesis in a sex-specific way	
	Sheep, gestational exposure to BPA	Increase in adipogenesis in a sex-specific way	Pu et al. [63]
		Proadipogenic effect is independent of PPAR*γ* activation	
	3T3 L1 cell culture	Proadipogenic effect stimulating glucocorticoid receptors (GR)	Sargis et al. [64], Boucher et al. [65]
		Proadipogenic effect via estrogens receptor (ER)	
		BPA-induced adipogenesis is inhibited by estrogen receptor (ER)	
	Human mesenchymal cells	Proadipogenic effect via SREBF1, TR/RXR, and mTOR	Boucher et al. [66]
	Human mesenchymal cells from omental tissue of children exposed to BPA	Increased expression of 11*β*-hydroxysteroid dehydrogenase type 1, PPAR*γ*, and lipoprotein lipase	Wang et al. [67]
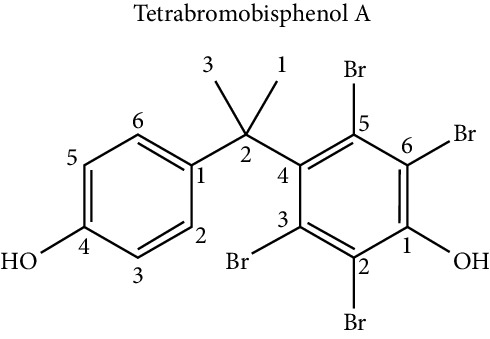	NIH3T3-L1	Proadipogenic effect via PPAR*γ*	Riu et al. [55]
	3T3 L1 cell culture	Proadipogenic effect in a dose-dependent manner via PPAR*γ*	Akiyama et al. [68]
	Human mesenchymal cells	Proadipogenic effect mediated by microRNA-103 induction	Woeller et al. [69]

Phthalates			
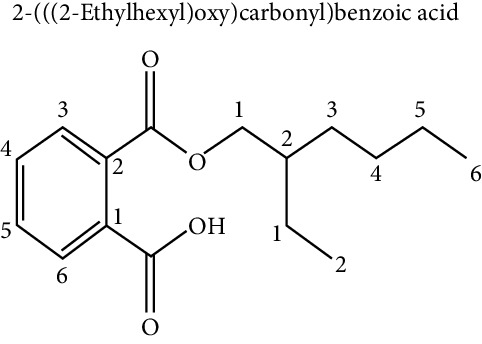	3T3 L1 cell culture	Proadipogenic effect via PPAR*γ*	Feige et al. [70], Bility et al. [71], Hao et al. [72]
	COS-1 cell culture	Activation of PPAR*γ*, PPAR*α*	Hurst and Waxman [54]
	Fetal exposure in mice	Increase in weight and fat mass in the offspring	Hao et al. [72]
	Human mesenchymal cells	Increase expression of transcripts related to the PPAR*γ* signaling pathway	Ellero-Simatos et al. [73]
		Increase expression of genes involved with lipid metabolism	

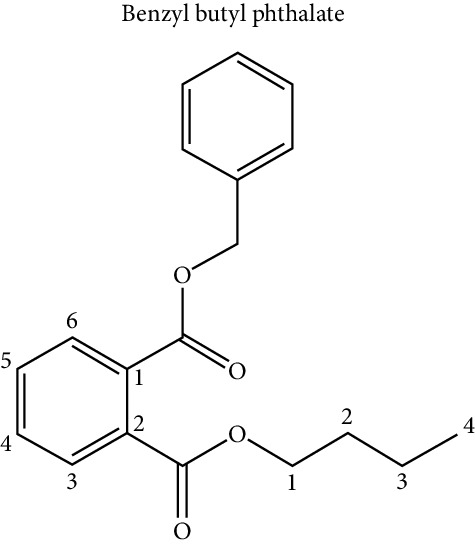	Bone marrow stromal cells	Decreased differentiation towards osteoblasts, and an increase in adipogenesis vía PPAR*γ*	Chiu et al. [74]
	3T3 L1 cell culture	Proadipogenic effect via PPAR*γ*	Yin et al. [75]
	Mouse mesenchymal cell lines C3H10T1/2.	Proadipogenic effect	Sonkar et al. [76]
		Epigenetic effect on histones of the PPAR*γ* promoter	

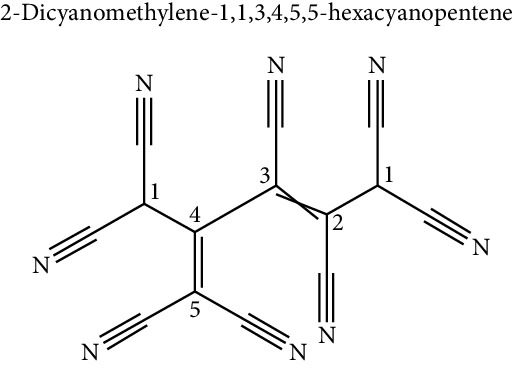	3T3 L1 cell culture	Proadipogenic effect via glucocorticoid receptor (GR)	Sargis et al. [64]

Triclosan			
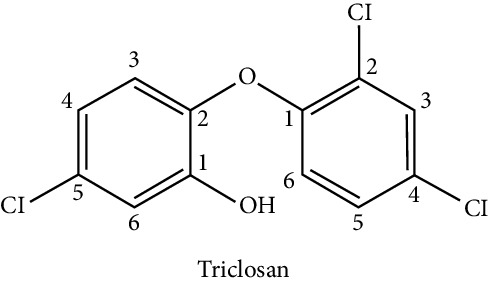	Human mesenchymal cells	Antiadipogenic effect	Guo et al. [77]
		Decrease of adiponectin and lipoprotein lipase	
PBDEs			
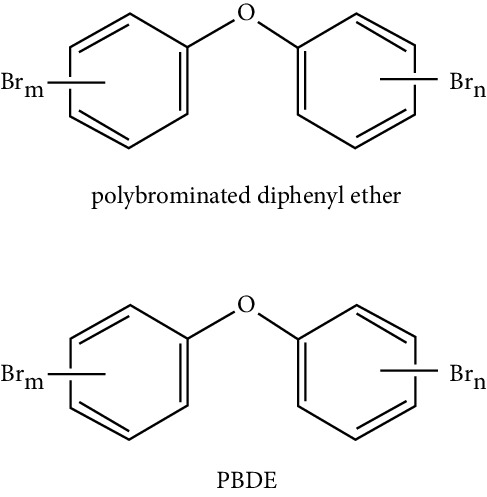	3T3 L1 cell culture	Proadipogenic effect via PPAR*γ*	Tung et al. [78]

Benzo-alpha-pyrene			
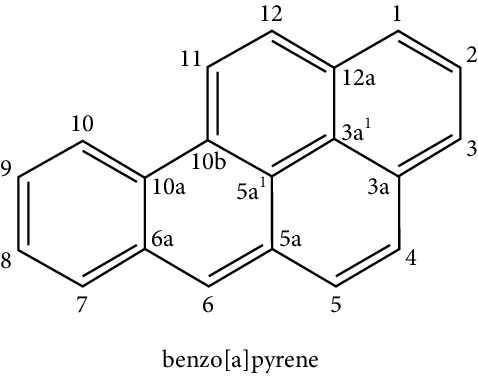	Human mesenchymal cells	Antiadipogenic effect via aryl-hydrocarbon receptor	Podechard et al. [79]
